# Cognitive behavioral therapy for lifestyle changes in patients with obesity and type 2 diabetes: a systematic review and meta-analysis

**DOI:** 10.1038/s41598-023-40141-5

**Published:** 2023-08-07

**Authors:** Katja Kurnik Mesarič, Jernej Pajek, Bernarda Logar Zakrajšek, Špela Bogataj, Jana Kodrič

**Affiliations:** 1 Department of Nephrology, University Medical Centre Ljubljana, Ljubljana, Slovenia; 2 Department of Psychology, Faculty of Arts, University of Ljubljana, Ljubljana, Slovenia; 3 Faculty of Medicine, University of Ljubljana, Ljubljana, Slovenia; 4 Management, Division of Surgery, University Medical Centre Ljubljana, Ljubljana, Slovenia; 5 Unit of Child Psychiatry, University Children’s Hospital, University Medical Centre Ljubljana, Ljubljana, Slovenia

**Keywords:** Human behaviour, Endocrine system and metabolic diseases, Obesity

## Abstract

The aim of this systematic review and meta-analysis was to examine the contribution of cognitive behavioral therapy (CBT) to the implementation of lifestyle changes, considering health-related and behavioral outcomes. A systematic literature review was performed using multiple databases (PsycInfo, PubMed and MEDLINE). The inclusion criteria comprised randomised controlled trials of CBT for lifestyle changes in patients with obesity and/or type 2 diabetes. The quality of study reporting was assessed with the revised Cochrane Collaboration’s risk of bias tool. A meta-analysis was conducted on studies with appropriate outcomes. Nine randomised controlled trials, with a total sample size of 902 participants, met the inclusion criteria. The meta-analysis has shown a medium, significant effect size of CBT interventions for weight loss and weight maintenance, and a low, non-significant effect size of CBT interventions for reducing glycated hemoglobin (HbA1c) levels. A separate, combined, meta-analysis for all nine calculated effect sizes has yielded a medium and significant overall effect size for the model. Our review of the studies about the effectiveness of CBT in implementing lifestyle changes has, in comparison to usual control groups, proven the efficacy of CBT interventions in implementing lifestyle changes, especially for weight loss and weight maintenance.

## Introduction

Chronic noncommunicable diseases are responsible for the majority of all deaths globally^1,2^. These diseases are the result of a combination of genetic, physiological, behavioral and environmental factors. The main types of chronic diseases are obesity, diabetes, cardiovascular diseases, cancers, chronic kidney diseases and respiratory diseases. The most common behavioral risks for chronic diseases are related to unhealthy food intake, physical (in)activity, alcohol use and tobacco use^1,3^. Lifestyle practices that play a key preventive role in health outcomes are regular physical activity, weight management, proper nutrition, avoiding tobacco use, quality sleep, and mental health/stress reduction^4^. The essential result of a healthy lifestyle is the maintenance of a normal body mass index (BMI).

Behavioral treatment contributing to lifestyle modifications along with multicomponent psychological interventions represent one of the core evidence-based treatment modalities in obesity and weight management, in addition to surgery and pharmacotherapy^5^. Behavioral treatment and cognitive behavioral therapy (CBT) are often used in weight loss counselling. The underlying assumptions of behavioral and CBT treatments are that behaviors can be learned, unlearned, modified, and replaced by other behaviors through different strategies, some of which include goal setting, problem solving, stimulus control and self-monitoring^6,7^.

CBT treatments can contribute to lifestyle modification, weight loss, and weight maintenance. Studies and reviews have been conducted in this area, and most recent reviews have shown the efficacy of CBT in weight loss^8,9^ as well as eating disorders^10^. The studies included in these reviews compared CBT treatments to other non-CBT active treatments (life-style interventions, behavioral treatment, education etc.) as well as control groups. To gain a clearer insight into the basic efficacy of CBT intervention, a comparison with non-active control groups was called for. It is a known fact that weight loss interventions have a very limited long-term efficacy^11^, which is why, in this systematic literature review, we have examined the effectiveness of CBT for implementing lifestyle changes with health-related and behavioral outcomes in patients with obesity and type 2 diabetes, including the efficacy in long-term maintenance of lifestyle modifications and the health benefits gained.

The aim of this systematic literature review was to examine the effectiveness of CBT in implementing lifestyle changes in patients with obesity and chronic diseases, focusing on health benefits achieved and long-term changes implemented. Although reviews have already been conducted in this area, there is still a research gap that requires further investigation, particularly to directly examine the effectiveness of CBT compared to non-active control groups.

Health-related outcomes included weight loss or changes in body mass index (BMI), changes in body mass index after active weight loss process (weight maintenance), and in glycated hemoglobin (HbA1c) levels. The examined behavioral outcomes were eating behaviors, physical activity, smoking, and treatment adherence in type 2 diabetes. Studies with suitable outcomes, based on adequate reporting, were subjected to a meta-analysis, followed by subgroup analysis which were performed in respect to the outcomes of individual studies classifies into categories of weight loss, weight maintenance, and diabetes control.

## Methods

### Literature search

**Figure 1:**
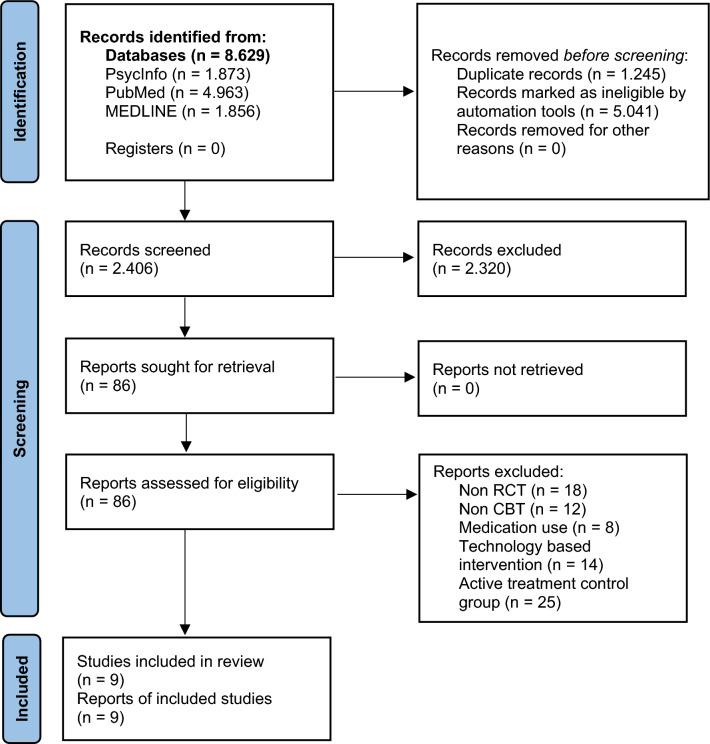
PRISMA flow diagram. *RCT* randomised controlled trial, *CBT* cognitive behavioral therapy.

The reviewing and reporting methods followed the Preferred Reporting Items in Systematic Review and Meta-Analyses (PRISMA) guidelines^12^. To identify potentially relevant studies, a comprehensive literature search was performed in electronic databases, including PsycInfo, PubMed, and MEDLINE (Ovid), from their inception to their final update in June 2022. Search strategies utilized a combination of keywords to represent definitions of CBT, lifestyle changes, obesity, type 2 diabetes, chronic kidney diseases, and other chronic diseases. The search terms were combined using the “AND” and “OR” Boolean operators (for the full list of search phrases and terms see Table 1 in Supplementary material). A flow diagram of the search is presented in Fig. 1.

### Screening procedures

#### Inclusion criteria

Studies were considered for inclusion a priori if they met the following criteria: (a) randomised controlled trials, (b) published in academic journals, (c) written in English, (d) adult participants aged 18 years or more, (e) with chronic noncommunicable diseases (type 2 diabetes, chronic kidney disease) and/or obesity and (f) studies with CBT interventions compared to usual or standard care (non-active control groups). Reviews, theses, abstracts, books and book chapters were excluded.

#### Title, abstract and full text screening

The process of reviewing the studies is documented in Fig. 1. The automation tools used in the databases were language (English), type of publication (Academic journals), type of study in the PubMed database (randomised controlled trial) and age of participants (adults) in the PsycInfo and PubMed databases. Articles from the databases were checked for duplicates using EndNote 20. After the removal of duplicates, the search resulted in 2.406 titles. Titles and abstracts were screened independently by two authors to identify studies that administered psychological interventions to promote lifestyle changes. After reviewing the titles and abstracts, 86 studies were retained for full text review. Full-texts of these articles were read to see whether the full inclusion criteria were met. All studies that met the inclusion criteria were re-screened independently by two authors to determine their eligibility for the systematic literature review.

### Risk of bias assessment

The quality of the included studies was evaluated using the revised Cochrane Collaboration’s risk of bias tool^13^. The following biases were evaluated: bias due to the randomization process, bias due to deviations from intended interventions, bias due to missing outcome data, bias in measurement of the outcome, bias in selection of the reported result, and overall bias. Risk of bias was assessed as low, some concerns, or high for each domain as well as for overall bias. Strength of evidence was determined using the Grades of recommendation, Assessment, Development, and Evaluation (GRADE)^14^.

### Data analysis

The analysis was performed using SPSS software, version 29. First, a random-effect meta-analysis with effect sizes (Glass’s Delta) was performed, followed by a calculation of the indicators of heterogeneity in the data with index I^2^. Publication bias was assessed with Egger’s regression test.

### Registration of protocol

The protocol for this systematic review was registered in the PROSPERO database under the registration number CRD42023444302.

## Results

### Study characteristics

Our original search targeted obesity, type 2 diabetes, and chronic kidney disease. Since we did not find corresponding papers for chronic kidney disease, we henceforth only focused on obesity and type 2 diabetes. The characteristics of the nine included studies are provided in Table 1. There was a total of 902 patients, 471 of which were allocated to the intervention group, with the rest (431) allocated to the control group. Participants in almost all the intervention groups (8 out of 9) received group CBT, using a wide range of behavioral and CBT strategies, and one of the studies compared self-help version of CBT to usual care^15^. The interventions spanned from 10 weeks to 1 year. The number of CBT sessions was between 3 and 17. Control groups were exposed to standard care with guideline handouts and brochures regarding a healthy diet and physical activity. Some studies also exposed controls to personal meetings with healthcare providers^16,17^ and some offered no specific treatment^18,19^. Interventions in active treatment groups included psychoeducation, self-monitoring, goal setting, problem solving, learning coping skills and strategies, stress management, relaxation, cognitive restructuring, and relapse prevention.

**Table 1:** Characteristics of included studies.

Study	Population and allocation	Intervention	Duration and number of sessions	Measures	Outcomes
Abdi et al.^20^	35 individuals with type 2 diabetes, HbA1c ≥ 7% BMI ≥ 25 kg/m^2^ I = 18 C = 17	Behavioral lifestyle intervention based on CBT, self-monitoring, goal setting for dietary changes, weight loss and physical activity Control—usual treatment	6 months 8 sessions	Diabetes control (HbA1c) Weight (BMI) Body composition Lipid profile Blood pressure Carbohydrate intake Minutes of exercise Medication adherence	Significantly reduced HbA1c levels in the intervention group and a reduced carbohydrate intake. A non-significant increase in the level of physical activity in the intervention group Difference between groups: I: Mean change in HbA1c after 6 months = − 1.17% C: Mean change in HbA1c after 6 months = − 0.17% ES: *Δ* = − 0.40
Berk et al.^21^	158 individuals with BMI ≥ 27 kg/m^2^ with type 2 diabetes I = 83 C = 75	Cognitive restructuration, working on core beliefs, goal setting, problem solving, proactive coping, relapse prevention Control—usual treatment	18 months 17 sessions	Weight (BMI) Waist circumference Lipid profiles Blood pressure HbA1c	CBT did not reduce weight regain following successful weight loss Difference between groups: I: weight regain after 1 year = 1.4 kg; after 2 years = 4.0 kg C: weight regain after 1 year = 1.7 kg; after 2 years 4.7 kg
Fernández-Ruiz et al.^22^	74 individuals with BMI ≥ 25 kg/m^2^ I = 37 C = 37	Psychoeducation, cognitive restructuring, problem solving, skills training Control—usual treatment	12 months 12 sessions	Weight (BMI) Body fat distribution Blood pressure	CBT group significantly reduced BMI; they lost 8.3% of body weight on average; control group gained 1% of body weight. Blood pressure improved greatly in the CBT group Difference between groups: I: Mean change in BMI after 1 year = − 2.6; after 2 years = − 2.7 C: Mean change in BMI after 1 year = − 0.1; after 2 years = + 0.3 ES: *Δ* (weight loss) = − 1.05 ES: *Δ *(maintenance) = − 1.20
Grilo et al.^15^	48 individuals with obesity (BMI ≥ 30 kg/m^2^) and binge eating disorder I = 24 C = 24	Self-help version of CBT: psychoeducation, self-monitoring, cognitive restructuring, coping skills, relapse prevention Control—usual treatment	4 months	Weight (BMI) Eating disorders Depression	Binge-eating remission rates did not differ significantly between groups. Weight loss was not observed in either condition Difference between groups: I: Mean change in BMI after 4 months = − 0.56 C: Mean change in BMI after 4 months = + 0.20 ES: *Δ* = 0.01
Madjd et al.^17^	113 women with BMI of 23 to 30 kg/m^2^ who had lost at least 10% of their body weight I = 56 C = 57	Weight maintenance diet and CBT: behavioral modification, goal setting, changing eating habits, time managements, problem solving, coping strategies, helpful beliefs, relapse prevention Control—weight maintenance diet only	6 months 10 sessions	Weight (BMI) Waist circumference Lipid profiles Glucose metabolism measurement	The CBT treatment group significantly improved weight maintenance, BMI and waist circumference, reduced energy intake and increased physical activity Difference between groups: I: Mean change in BMI after 6 months = − 0.43 C: Mean change in BMI after 6 months + 0.23 ES: *Δ* = − 0.48
Safren et al.^23^	87 individuals with type 2 diabetes and depression I = 45 C = 42	Cognitive restructuring, problem solving, mood monitoring, increasing pleasurable activities, behavioral activation, relaxation training Control—usual treatment	4 months 9–12 sessions	Medication adherence Adherence to glucose monitoring Diabetes control Depression	The CBT group had better medication and glucose monitoring adherence as well as significantly lower levels of HbA1c after treatment. These effects were maintained over 8 months of follow-up. The CBT group also had lower depression scores after treatment Difference between groups: I: Mean change in HbA1c after 4 months = − 0.95% C: Mean change in HbA1c after 4 months = − 0.16% ES: *Δ* = − 0.69
Sallit et al.^19^	128 women with BMI between 24 and 43 kg/m^2^ and smoking I = 70 C = 58	Self-monitoring, goal setting, stimulus control, cognitive restructuring, challenging and modifying unrealistic thoughts, stress management Control—no treatment	3 months 12 sessions	Weight (BMI) Eating disorders Eating behaviors Smoking related measures	Significant weight loss in the intervention group, improved diet quality, decreased number of smoked cigarettes, increased self-efficacy for quitting smoking and for weight control Difference between groups: I: Mean change in BMI after 3 months = − 1.64; after 1 year =− 0.95 C: Mean change in BMI after 3 months = − 0.42; after 1 year = + 0.24 ES: *Δ* (weight loss) = − 0.59 ES: *Δ* (maintenance) = − 0.51
Stahre and Hällström^18^	105 individuals with obesity (BMI ≥ 30 kg/m^2^) I = 62 C = 43	Psychoeducation, self-control, stress management, changing eating behaviors, identifying thinking and behavior patterns, identifying emotions Control—no treatment	10 weeks 10 sessions	Weight (BMI) Eating disorders	Significant weight loss in the intervention group (average weight loss after treatment was 8.5 kg and 10.4 kg 18 months after treatment), the control group gained weight during the same period Difference between groups: I: weight change after 18 months = − 10.4 kg C: weight change after 18 months = + 2.3 kg
Welschen et al.^16^	154 individuals with type 2 diabetes and Hba1c ≥ 7% and/or BMI ≥ 27 kg/m^2^ and/or smoking I = 76 C = 78	Managed diabetes care and CBT focused on problem solving treatment Control—usual treatment	6 months 3 to 6 CBT sessions	CHD risk Physical activity Quality of life Depression Determinants of behavior change	The intervention group significantly increased their physical activity and improved their quality of life as well as reduced depression symptoms. After 6 months, all effects disappeared Difference between groups: I: Mean change in HbA1c after 4 months = – 0.1% C: Mean change in HbA1c after 4 months = 0.0% ES: *Δ* = 0.00

*I* intervention group, *C* control group, *CBT* cognitive behavioral therapy, *BMI* body mass index, *HbA1c* glycated hemoglobin, *ES* effect size, *Δ* Glass's delta.

The primary outcome of interest (or, in some cases, secondary) in the studies was weight or BMI. Other outcomes measured in the studies were diabetes control (HbA1c level), body composition, lipid profile, blood pressure, weight control behaviors, health related behaviors (physical activity, smoking, adherence to medication, carbohydrate intake etc.) and psychological variables (depression, severity of eating disorder features).

### Study quality

**Figure 2:**
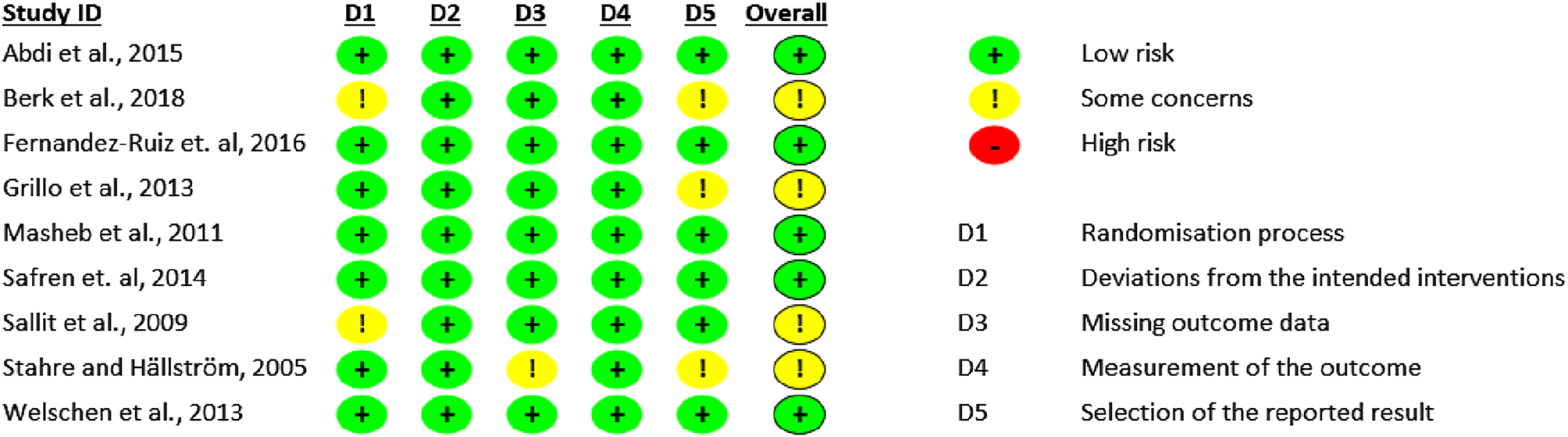
Risk of bias summary: review authors’ judgements about each risk of bias item for every included study.

The risk of bias is summarised in Fig. 2. All studies performed well regarding deviations from the intended interventions and measurements of the outcomes. There were some concerns regarding randomization, missing outcome data and selection of the reported results^15,18,19,21^. Certainty of evidence assessment is summarised in Table 2 in Supplementary material. Overall, studies have performed well.

### Health related outcomes

In most of the studies included in this review (where weight loss was the goal of the intervention), the interventions were found to have had at least some effect on weight loss^17–19,22^. In two of the studies, the intervention had no effect on weight loss^15,16^. Three of the included studies reported data for weight change in BMI immediately after the weight loss intervention.

**Figure 3:**
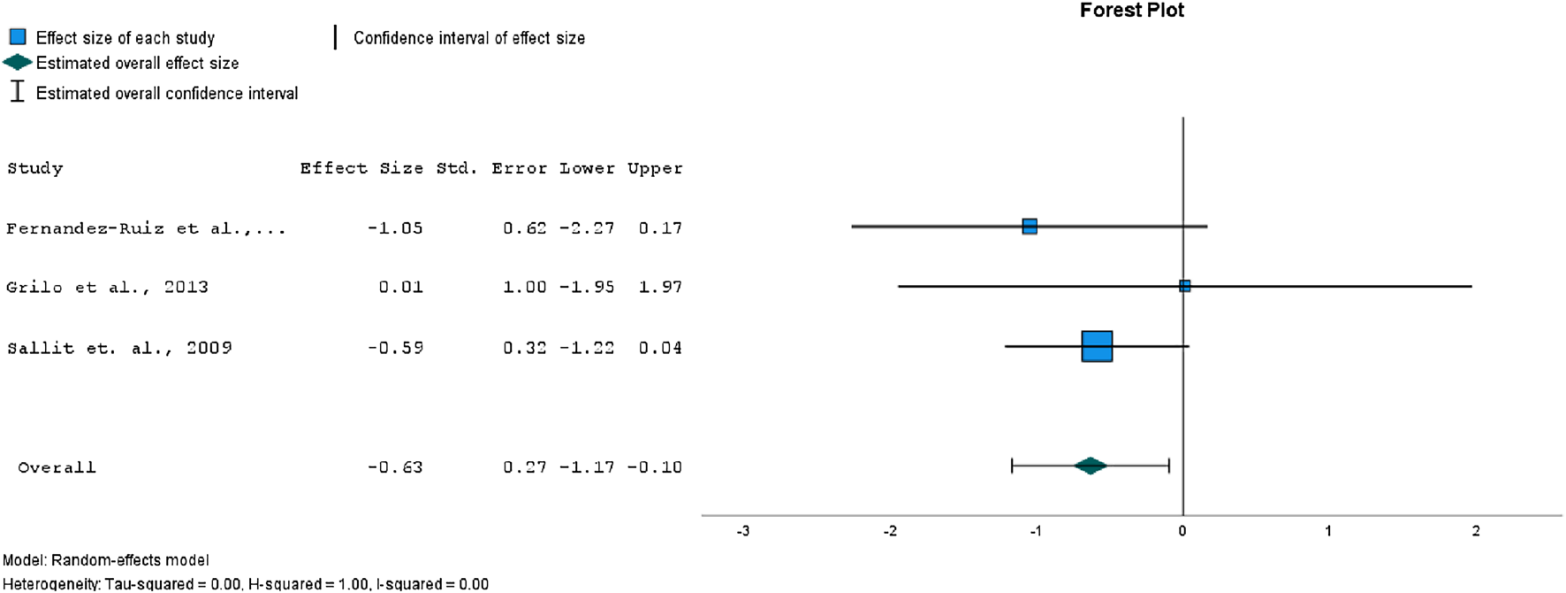
Forest plot comparing effect sizes between intervention and control groups, outcome: weight loss (expressed as BMI change).

The forest plot for weight loss expressed as BMI change in Fig. 3 has shown a medium range significant effect size (*p* = 0.02) of CBT interventions. There was evidence of low heterogeneity (*I*^2^ = 0%). Egger’s test for a regression intercept did not indicate publication bias (*p* = 0.463). The mean BMI change in the weight loss phase was − 1.6 in the intervention groups, and -0.05 in the control groups.

One of the biggest challenges in weight control is regaining weight after an initial loss. CBT treatment may also improve maintenance of weight loss^17,18^, but this has not been the case in all studies examining the long-term effects^21^.

**Figure 4:**
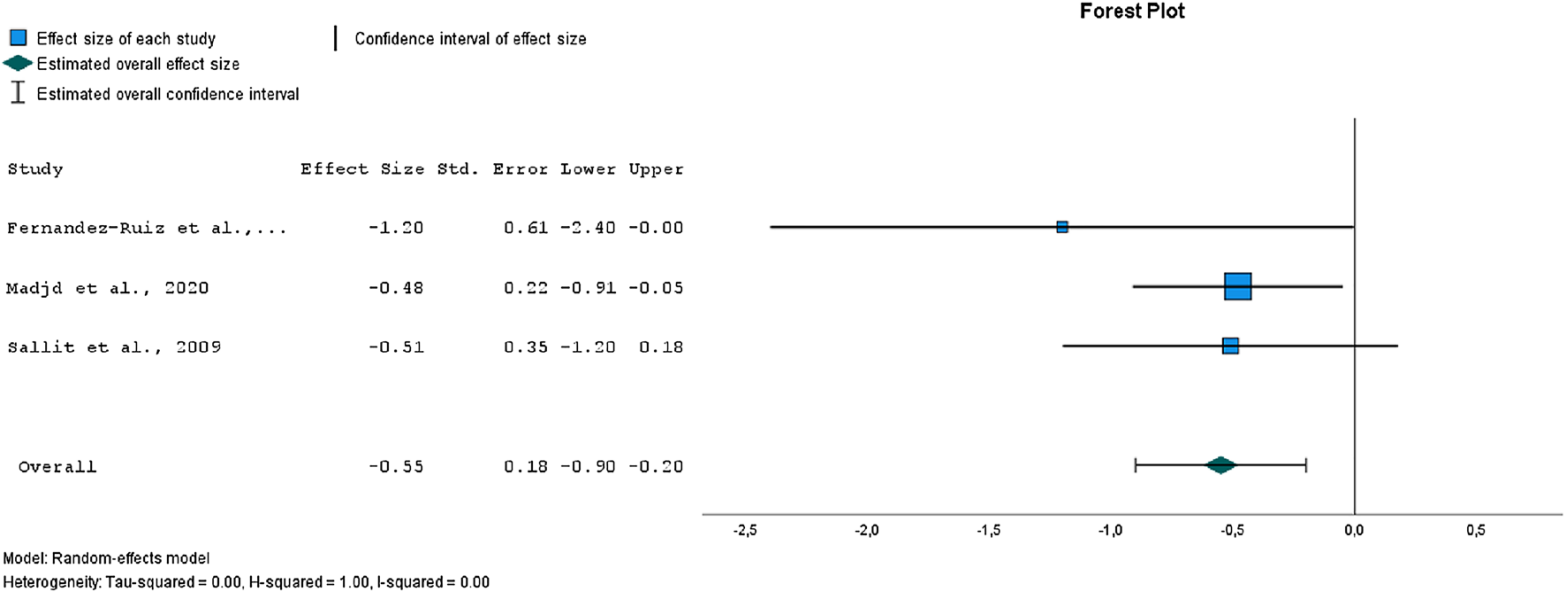
Forest plot of comparison of effect sizes between intervention and control groups, outcome: weight maintenance (BMI).

The forest plot in Fig. 4 has shown a medium range significant effect size (*p* = 0.002) for weight maintenance. There was evidence of low heterogeneity (*I*^2^ = 0%). Egger’s test for a regression intercept did not indicate publication bias (*p* = 0.876). The mean BMI change in the weight maintenance phase was − 1.36 in the intervention groups, and 0.26 in the control groups.

Three studies^16,20,23^ used CBT interventions for improving type 2 diabetes control. Compared to the control group, there were 2 studies with a significant (intra-group) effect on reducing HbA1c levels in patients with type 2 diabetes, with the effect of significant reduction persisting after one year^20,23^. In one of the studies, no significant effect (intra-group) on HbA1c level was observed^16^.

**Figure 5:**
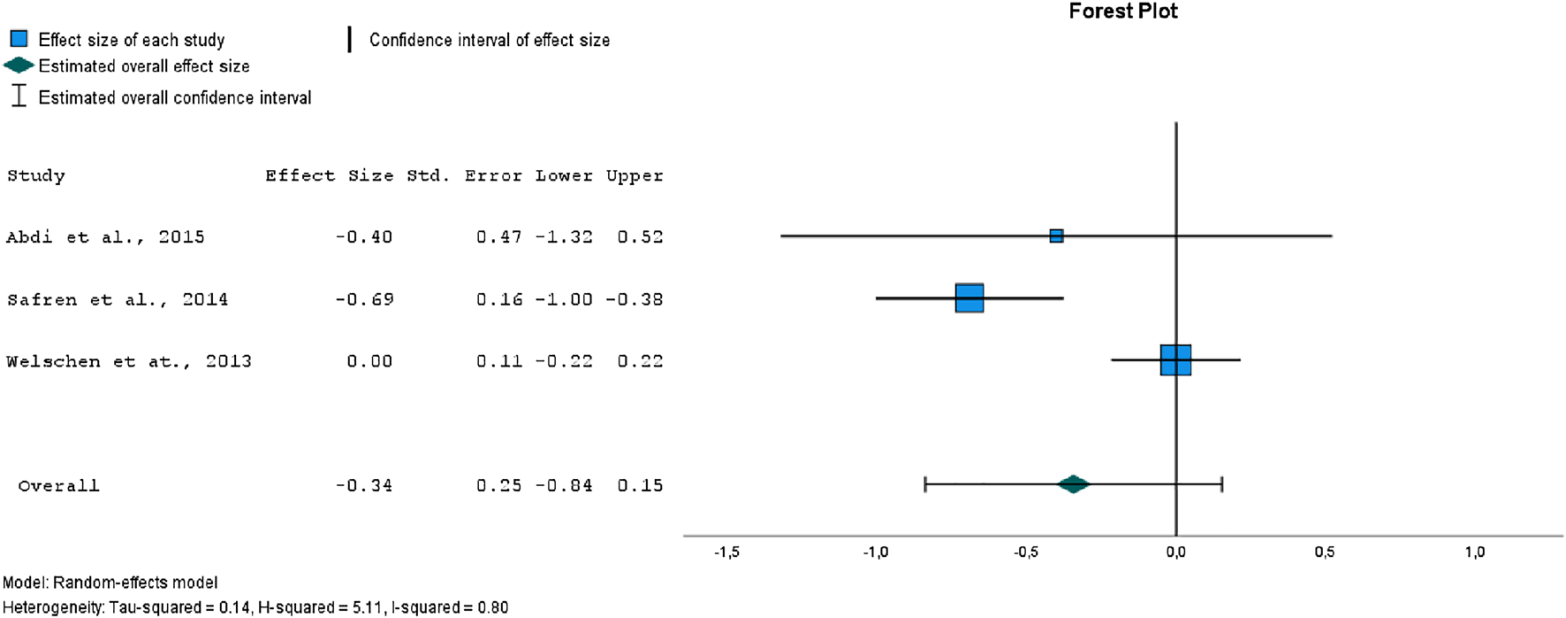
Forest plot of comparison of effect sizes between intervention and control groups, outcome: HbA1c levels.

The forest plot in Fig. 5 has shown a low range non-significant (*p* = 0.17) effect size for reduction of HbA1c levels. There was evidence of substantial heterogeneity (*I*^2^ = 80%). Egger’s test for a regression intercept did not indicate publication bias (*p* = 0.175).

**Figure 6:**
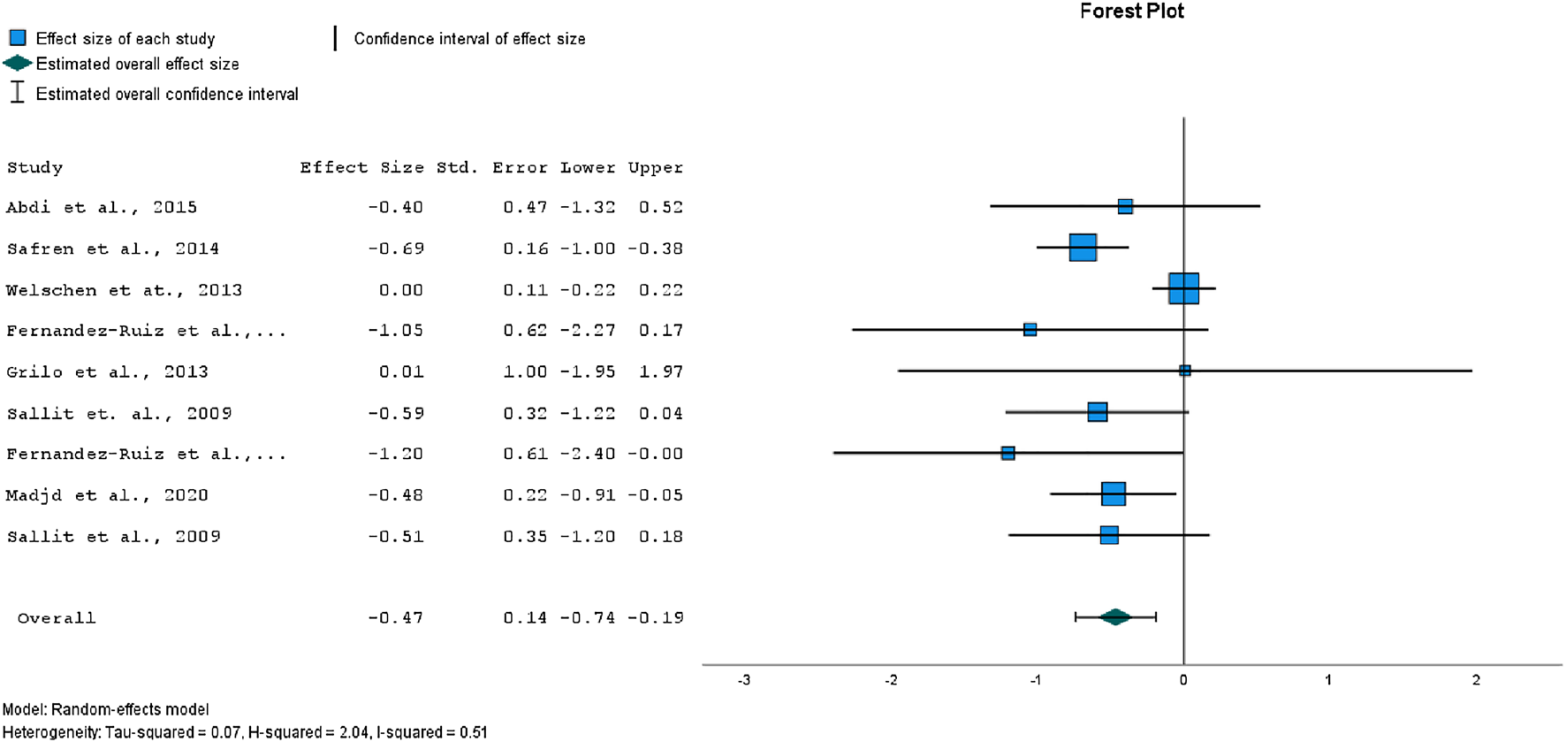
Forest plot of comparison of effect sizes for all health-related outcomes.

A separate meta-analysis was performed for all nine studies mentioned above, with their calculated effect sizes combined. Figure 6 shows a medium and significant overall effect size for the model (*p* < 0.001). There was evidence of medium heterogeneity (*I*^2^ = 50%). Egger’s test for a regression intercept did not indicate publication bias (*p* = 0.354).

### Behavioral and psychological outcomes

Due to the heterogeneity of outcomes, we have reviewed the behavioral and psychological outcomes in a descriptive manner only. CBT intervention successfully helped to improve diet quality in a single study^19^. CBT interventions also helped to reduce energy intake^17^ and the selection of carbohydrates from inferior sources (reduction of carbohydrate intake form cereals and dairy products^20^).

Patients with type 2 diabetes who participated in CBT treatment reached better medication adherence (16.3% higher medication adherence in the CBT group, compared to the control group; *p* = 0.001) and self-monitoring of blood glucose levels (22.3% better glucose monitoring adherence in the CBT group; *p* = 0.002^23^).

CBT treatment led to a significant increase in physical activity among participants—there was a significant increase in daily steps in the CBT group^17^ and an increase in moderate (intervention group 51 min, control group 30 min) and heavy (intervention group 23 min, control group 17 min) daily activities^16^. In one of the cases, the increase in physical activity was not significant, but the results indicated increased physical activity in the intervention group (baseline 4.67 ± 9.98 min of exercise per day, after the intervention 9.12 ± 17.39 min per day^20^).

CBT also influenced smoking cessation. Compared with the control group, participants in the intervention group smoked fewer cigarettes per day (12.66 versus 15.76) and had a greater intention to quit smoking, measured with the Smoking Self-Efficacy Questionnaire^19^.

Depression outcomes were examined in three studies. In all of them, the CBT intervention resulted in a significant reduction in depression scores after treatment (mean score on the Beck Depression Inventory before treatment 14.57 ± 8.48, mean score on the BDI after treatment 8.88 ± 7.67^15^; mean score on the Montgomery-Asberg Depression Rating Scale before treatment 25.60, mean score after treatment 14.83^23^; mean score on the Center for Epidemiologic Studies Depression Scale before treatment 11.1 ± 8.1, mean score after treatment 9.9 ± 7.7, significant difference at the 0.05 level^16^.

## Discussion

### Summary of evidence

The aim of this systematic literature review and meta-analysis was to evaluate the effectiveness of CBT interventions for lifestyle changes compared to inactive control groups, including both the efficacy at induction and the weight maintenance phase. A total of 9 studies were analysed involving 902 patients with obesity and/or type 2 diabetes. The studies have shown the efficacy of CBT approaches in several areas and the meta-analysis has demonstrated a statistically significant effect size of the CBT interventions for weight loss and weight maintenance. For diabetes control, results have shown the efficacy of CBT interventions; however, a statistically significant effect size could not be demonstrated. For all outcomes combined, the effect of the CBT interventions was significant and had an effect size in medium range.

#### Health related outcomes

Regarding short-term weight loss, our meta-analysis has found a moderate significant effect of CBT interventions, with three studies successfully demonstrating the effectiveness of CBT^18,19,22^. In two of the studies, the interventions did not result in weight loss; in the first case, the intervention was self-help CBT^15^, and the lack of sessions with a therapist may have contributed to the outcome. Another limitation of the study was that the patients did not report adherence to the self-help programme, making it difficult to determine whether patients had read and followed the programme. In the second study with an ineffective intervention^16^, the intervention was delivered by nurses who had only completed two days of CBT training, which may have contributed to the limited results of the intervention. Studies have shown that both the therapeutic alliance and therapist competence are related to therapy outcomes^24,25^.

Obesity is related to psychological variables, and therefore psychological and psychotherapeutic interventions are one of the key elements in the management of obesity^26^. For obesity, CBT interventions are the therapy of choice^27^. Two recent meta-analyses have shown the efficacy of CBT for weight loss^9,28^, especially for increasing cognitive restraint and reducing emotional eating, but the effect size was small^9^. Our review, limited to the comparison of active treatment to inactive controls, has shown a similar result; however, we were only able to find an effect of CBT interventions of a moderate size.

CBT is one of the most studied forms of psychotherapy and it is known to be effective for various (mental) health problems^29,30^. The CBT interventions used in the studies included a variety of strategies used in CBT treatments. One of the most important common features of the interventions was the use of self-monitoring, followed by goal setting, working with (unrealistic) thoughts (cognitive restructuring, working on core beliefs), problem-solving strategies, and relapse prevention. The use of self-monitoring in weight loss originates from behavioral approaches to weight loss and it represents a core procedure for implementing lifestyle modifications^31^. Behavioral approaches alone have proven not to be efficient enough, as they tend to overlook the role of cognitive processes in weight loss^32^. The most commonly used cognitive strategies in the included studies were working with unrealistic thoughts, cognitive restructuring, and working on core beliefs. To achieve better efficacy CBT approaches integrate behavioral and cognitive strategies.

Patients often successfully lose weight, but the main challenge remains maintaining the lost weight^11^. Regarding weight maintenance, our meta-analysis has shown a moderate, significant effect of the CBT interventions. In all of the studies that provided sufficient data on weight maintenance, participants in the intervention groups were more successful in maintaining weight loss compared to the control group^17,19,22^.

One of the main health outcomes of interest for us was diabetes glycemia control (measured with HbA1c levels). The meta-analysis has shown a small, nonsignificant effect size in lowering HbA1c. There was a significant reduction in HbA1c in two of the three studies^20,23^, and no reduction in HbA1c in the third study^16^. As mentioned earlier, the intervention in this study was delivered by nurses who had only completed two days of CBT training, and a lack of therapist competence may contribute to ineffective interventions^24,25^.

#### Behavioral outcomes

CBT can contribute to behavioral lifestyle changes, as one of the main focuses of CBT is learning new, more appropriate behaviors^6^. Studies included in this review have addressed many different behavioral outcomes, so performing a combined meta-analysis was not appropriate. Descriptive analysis of included studies has shown that CBT may help with improving the quality of diet, the level of physical activity and smoking cessation^17,19,20^. The dataset, however, is small.

Chronic diseases, such as type 2 diabetes, require adjustments in many aspects of daily life, e. g. healthy eating, physical activity, and adherence to diabetes management medications and instructions. CBT has been recognised as a type of psychotherapy suitable for the management of glycaemic control^33^. In two of the three studies included in our review in which adherence was of interest, CBT was found to be beneficial for adherence to diabetes management instructions^20,23^. The most recent study published in this field shows beneficial results of CBT intervention in patients with type 2 diabetes, as patients in the intervention group had significantly lower diabetes distress, improved treatment adherence, and increased physical activity^34^.

CBT treatment may also improve depression scores, which is not surprising, given that CBT is one of the most evidence-based psychological interventions for psychiatric disorders such as depression and anxiety, and it is offered as first in line for mild to moderate depression^35^. Our review included three studies that had examined the effects of the intervention on depression^15,16,23^. All have shown a significant reduction in depression scores after the intervention, although the intervention did not target depression. Components of CBT interventions aimed at weight loss (e. g. physical activity, goal setting, cognitive restructuring) can also contribute to mood improvement. Loss of interest is one of the key aspects of depression. With monitoring and planning (which are both components of CBT interventions for weight loss), we can provide structure, help with setting priorities, and increase the patients’ sense of control and autonomy^35^. Another key component of CBT for depression is working with negative automatic thoughts, which is also covered in CBT interventions for weight loss.

### Limitations

The established inclusion criteria for the literature search, were relatively strict. A key criterion and the one yielding the most exclusion was the presence of a comparative control group with no treatment, or usual standard care treatment without a specific active psychological intervention. This gave us much needed insight into the extent of basic effectiveness of CBT treatments, but on the other hand, severely limited the number of included studies. As seen in Fig. 1, 25 studies have been excluded based on these criteria, since these studies compared CBT to other active treatments such as behavioral therapy and other psychotherapeutic or general lifestyle interventions.

One of the main limitations of the included studies was the relatively small sample size in many of the studies^15,20,22,23^. Additionally, there were two studies with only female participants^17,19^. Almost all of the studies used self-reporting of some behavioral measures and outcomes, and it is a known fact (for example in the case of physical activity) that people have the tendency to exaggerate in these self-reports^16^. Due to the fact that the subjects have to actively participate and be in contact with the therapist, CBT intervention studies lack blinding, which can be another drawback of studies in this field.

Last but not least, some of the studies^18–20^ did not explicitly report a statistical between-group comparison of the outcomes, which makes it hard to perform a qualified judgement of the intervention effectiveness.

### Further directions

Future research evaluating CBT treatment for weight loss and maintenance should employ CBT interventions tailored specifically for obesity management (e.g., Dalle Grave et al.^32^). The study design of the randomised controlled trials should include a sufficiently large sample of participants of both genders. It would be useful to investigate the duration of an effective CBT intervention. Any study done should focus not only on short-term weight loss (induction phase) but, crucially, also on long-term weight maintenance. Given the poorer outcomes of the included studies, which didn’t employ specialised professionals, we propose that the intervention is delivered by a certified CBT therapist with training in obesity management.

## Conclusions

Our review of studies on the efficacy of CBT in lifestyle modification compared with the usual-care control groups shows moderate efficacy of CBT interventions in treating obesity and implementing lifestyle changes. Regarding diabetes control, there is a need for future research to draw firmer conclusions about the effectiveness of CBT interventions.

### Supplementary Information

Supplementary Tables.

## Data Availability

The studies included in this review will be available from the corresponding author.
