# Author Correction: Cognitive behavioral therapy for lifestyle changes in patients with obesity and type 2 diabetes: a systematic review and meta-analysis

**DOI:** 10.1038/s41598-023-49838-z

**Published:** 2023-12-19

**Authors:** Katja Kurnik Mesarič, Jernej Pajek, Bernarda Logar Zakrajšek, Špela Bogataj, Jana Kodrič

**Affiliations:** 1 Department of Nephrology, University Medical Centre Ljubljana, Ljubljana, Slovenia; 2 Department of Psychology, Faculty of Arts, University of Ljubljana, Ljubljana, Slovenia; 3 Faculty of Medicine, University of Ljubljana, Ljubljana, Slovenia; 4 Management, Division of Surgery, University Medical Centre Ljubljana, Ljubljana, Slovenia; 5 Unit of Child Psychiatry, University Children’s Hospital, University Medical Centre Ljubljana, Ljubljana, Slovenia

Correction to: *Scientific Reports* 10.1038/s41598-023-40141-5, published online 07 August 2023

The original version of this Article omitted an affiliation for Katja Kurnik Mesarič and for Jernej Pajek, respectively. Their correct affiliations are listed below.

Katja Kurnik Mesarič:

Department of Nephrology, University Medical Centre Ljubljana, Ljubljana, Slovenia.

Department of Psychology, Faculty of Arts, University of Ljubljana, Ljubljana, Slovenia.

Jernej Pajek:

Department of Nephrology, University Medical Centre Ljubljana, Ljubljana, Slovenia.

Faculty of Medicine, University of Ljubljana, Ljubljana, Slovenia.

The original Article has been corrected.

