# Supplementary material for: Cognitive behavioral therapy for lifestyle changes in patients with obesity and type 2 diabetes: a systematic review and meta-analysis
Source: Sci Rep. 2023 Aug 7;13:12793. doi: 10.1038/s41598-023-40141-5 (PMC10406954; doi:10.1038/s41598-023-40141-5)
Supplement: Supplementary file 1 — Supplementary Tables. [file 41598_2023_40141_MOESM1_ESM.docx]

**Supplementary material**

Table 1. Search strategy

| Literature search | |
| --- | --- |
| PsycInfo | cognitive behavioral therapy or cbt AND diabetes type 2 or diabetes mellitus type 2 or diabetes 2 |
|  | cognitive behavioral therapy or cbt AND ckd or chronic kidney disease |
|  | cognitive behavioral therapy or cbt AND obesity |
|  | cognitive behavioral therapy or cbt AND lifestyle changes or lifestyle modification or lifestyle intervention |
|  | cognitive behavioral therapy or cbt AND lifestyle changes or lifestyle modification or lifestyle intervention AND diabetes type 2 or diabetes mellitus type 2 or diabetes 2 |
|  | cognitive behavioral therapy or cbt AND lifestyle changes or lifestyle modification or lifestyle intervention AND ckd or chronic kidney disease |
|  | cognitive behavioral therapy or cbt AND lifestyle changes or lifestyle modification or lifestyle intervention AND obesity |
|  | cognitive behavioral therapy or cbt AND lifestyle changes or lifestyle modification or lifestyle intervention AND chronic illness or chronic disease |
|  | cognitive behavioral therapy or cbt AND chronic illness or chronic disease |
| Filters | english, academic journals, adult population |
| PubMed | cognitive behavioral therapy [Mesh] AND diabetes type 2 |
|  | cognitive behavioral therapy [Mesh] AND obesity |
|  | cognitive behavioral therapy [Mesh] AND chronic kidney disease |
|  | cognitive behavioral therapy [Mesh] AND lifestyle [Mesh] |
|  | cognitive behavioral therapy [Mesh] AND chronic disease |
|  | cognitive behavioral therapy [Mesh] AND lifestyle intervention |
|  | cognitive behavioral therapy [Mesh] AND lifestyle modification |
|  | cognitive behavioral therapy [Mesh] AND lifestyle change |
| Filters | english, randomised controlled trials, adult population |
| Medline | cognitive behavioral therapy AND diabetes type 2 |
| (OVID) | cognitive behavioral therapy AND kidney disease |
|  | cognitive behavioral therapy AND Renal Insufficiency, Chronic |
|  | cognitive behavioral therapy AND lifestyle |
|  | cognitive behavioral therapy AND obesity |
|  | cognitive behavioral therapy AND chronic disease |
| Filters | english, academic journals, expand term finder |

Table 2. Grading of Recommendations Assessment, Development and Evaluation (GRADE9 assessment details by confidence domain: risk of bias, non-reporting bias, imprecision, inconsistency, and indirectness

| Outcome | Number of studies | Design of studies | Risk of bias | Inconsistency | Indirectness | Imprecision | Publication bias | Effect size | Certainty of evidence |
| --- | --- | --- | --- | --- | --- | --- | --- | --- | --- |
| Weight loss | 3 | RCT | Not serious | Not serious | Not serious | Not serious | Undetected | -0,63  (95% CI from -1,17 to -0,10) | High |
| Weight maintenance | 3 | RCT | Not serious | Not serious | Not serious | Not serious | Undetected | -0,55  (95% CI from -0,90 to -0,20) | High |
| Glycated haemoglobin change | 3 | RCT | Not serious | Not serious | Not serious | Not serious | Undetected | -0,34  (95% CI from -0,84 to 0,15) | High |
| Lifestyle changes | 9 | RCT | Not serious | Not serious | Some | Not serious | Undetected | -0,47  (95% CI from -0,74 to -0,19) | Moderate |

RCT = randomized controlled trial, CI = confidence interval
